# Heavy Pigs Reared for Italian Dry-Cured Products: Does Immunocastration Influence the Fatty Acid Profile of Loins and Backfat?

**DOI:** 10.3390/ani14091284

**Published:** 2024-04-24

**Authors:** Marta Comin, Gaia Pesenti Rossi, Lydia Lanzoni, Paraskevi Prasinou, Annalaura Lopez, Giorgio Vignola, Sara Barbieri, Emanuela Dalla Costa

**Affiliations:** 1 Dipartimento di Medicina Veterinaria e Scienze Animali, Università degli Studi di Milano, 26900 Lodi, Italy; marta.comin@unimi.it (M.C.); gaia.pesenti@unimi.it (G.P.R.); sara.barbieri@unimi.it (S.B.); emanuela.dallacosta@unimi.it (E.D.C.); 2 Facoltà di Medicina Veterinaria, Università degli Studi di Teramo, 64100 Teramo, Italy; llanzoni@unite.it (L.L.); pprasinou@unite.it (P.P.); gvignola@unite.it (G.V.)

**Keywords:** heavy pig, carcass traits, fat quality, fatty acids, immunocastration

## Abstract

**Simple Summary:**

The Italian pig sector is renowned for dry-cured ham production, which requires heavy pigs (raised for at least nine months and slaughtered at more than 160 kg). Nevertheless, efficiency and utilisation of every part of the pig, including the loin, should be maximised. To avoid boar taint, surgical castration is necessary, albeit raising welfare concerns. Immunocastration offers a promising alternative. This study investigated the effects of immunocastration compared to surgical castration on the chemical composition and fatty acid profile of loins and adipose tissue from Italian heavy pigs. The results revealed differences in the fatty acid profiles, suggesting that immunocastrated meat may offer higher levels of saturated fatty acids. These findings highlight the importance of exploring alternative castration methods in terms of pig welfare improvement while also considering meat quality.

**Abstract:**

The Italian pig sector requires heavy pigs (raised for at least nine months and slaughtered at >160 kg). In order to avoid boar taint and lower the impact on welfare, immunocastration provides an alternative to surgical castration. This study investigated the effects of immunocastration compared to surgical castration on the chemical composition and fatty acid profile of loins (*longissimus dorsi* muscle) and adipose tissue in Italian heavy pigs raised for dry-cured ham. Twenty-four male pigs were subjected to surgical castration (*n* = 12) or immunocastration (*n* = 12). Carcass parameters were monitored at slaughter, and samples of *longissimus dorsi* muscle and subcutaneous fat were analysed. This study showed no significant differences in carcass characteristics and proximate composition of fresh meat. However, variations were observed in the fatty acid profiles of meat and adipose tissue between groups. Notably, saturated fatty acids, particularly stearic acid (18:0), were higher in the intramuscular fat (IMF) of the immunocastrated pigs compared to the surgically castrated pigs. Conversely, monounsaturated fatty acids, predominantly oleic acid (18:1n-9), were higher in the IMF from the surgically castrated pigs compared to the immunocastrated pigs. While immunocastration may offer benefits in terms of animal growth and carcass composition, it could lead to unfavourable lipid changes in fresh loin meat for Italian heavy pigs.

## 1. Introduction

The process leading to the production of pork products, especially hams, in Italy follows a traditional approach and involves the use of meat with an excellent aptitude for salting and curing for preparations [1]. A peculiarity of the Italian pork-based production system is the use of heavy pigs, characterised by high body weights and age at slaughter (approximately 160–180 kg and over 9 months of age). For instance, one of the Italian excellences in the European agricultural food production, the dry-cured Parma ham, should satisfy the strict regulations and standards set by the Italian ham industry to obtain the Protected Designations of Origin (PDO) label (Reg. (EC) No 148/2010). Particularly, the fresh-trimmed hind legs used to produce ‘Prosciutto di Parma’ should preferably weigh between 12 and 14 kg but must never weigh less than 10 kg. Thus, the weight of pigs slaughtered for this production is approximately 160 kg. Moreover, the intended specific characteristic to produce cured hams should not negatively affect the quality of the meat and backfat for the fresh pork market.

A common important issue found in both dry-cured and fresh pork products is the need to reduce the occurrence of boar taint in the meat [2,3] caused by the accumulation of androstenone and skatole in the fat of entire males. Castration of male piglets is the commonly used method to prevent the risk of this unpleasant characteristic through the reduction in androstenone and skatole contents in the fat tissue [4]. The characteristics of carcasses from castrated pigs present several differences compared to carcasses from uncastrated pigs [5]; particularly, the former tend to be fatter [6,7], and fat can contribute to the flavour and juiciness of the meat. Additionally, castrated pigs may have reduced growth rates and poorer feed-conversion ratios compared to entire males, which can lead to reduced profitability for pig producers [8].

Considering the age at slaughter and the need for excellent-quality thighs, castration is an unavoidable practice for rearing Italian heavy pigs intended for PDO hams [9]. For the Italian pork industry, surgical castration, besides preventing boar taint of the meat and to increase carcass fat, is also fundamental to reduce the occurrence of aggressive behaviours [10,11]. However, castration, normally performed without anaesthesia and analgesia, is a painful procedure that can cause short-term pain and discomfort and may also lead to long-term behavioural and health problems [12]. Consequently, surgical castration of pigs can have negative impacts on animal welfare and production characteristics when compared to leaving male pigs intact. The growing public concern and attention to animal welfare issues in pig production has led to a search for more welfare-friendly alternatives than surgical castration of male pigs to keep the unpleasant boar taint under control. In heavy pig farming, particularly considering that raising entire males is not an option, immunocastration has emerged as a practical alternative to surgical castration.

Immunocastration is a procedure that causes immunisation against gonadotropin releasing hormone (GnRH), a hormone produced in the hypothalamus that induces the secretion of luteinising hormone (LH) and follicle-stimulating hormone (FSH) by the anterior pituitary gland. These two gonadotropins act upon the gonads to stimulate testis growth and steroidogenesis in the males [13]. The procedure induces the suppression of the hypothalamic–pituitary–gonadal axis, which subsequently leads to the regression of testicular function and, therefore, to a drop in the concentration of hormones responsible for boar taint [14,15]. The procedure consists of two subcutaneous administrations: the first, carried out between 8 and 12 weeks of age, sensitises the animal towards GnRH, while the second, carried out between 6 and 4 weeks before slaughter, leads to the effective immune reaction against the hormone and the above-mentioned effects [16]. Since the effect of vaccination is temporary, an Italian study highlighted the need for a third administration to produce heavy pigs [17]. The effects of immunocastration on carcass traits, meat quality parameters, and fatty acid profile have been investigated by several authors [6,7,8,17,18,19,20,21,22,23,24]. Overall, immunocastrated pigs grow faster with a heavier carcass and show a lower fat thickness and a higher percentage of lean meat when compared with surgically castrated pigs [8,19,20,22]. Regarding information on the fatty acid profile for pigs intended for the dry-cured production process, literature data suggest a possible altered fatty acid composition [8,18,19,20,21,23,24] with small variations due to the difference in backfat depth [23]. However, the differences identified do not appear to negatively affect the suitability of the leg to enter the dry-cured ham production process [8,20,23]. To date, only one study has dealt with typical Italian heavy pigs reared for dry-cured ham production [17]. The results from that study revealed that, with a three-dose treatment, the difference between immunocastrated and surgically castrated pigs tends to decrease, resulting in an equivalent meat quality of dry and green hams in males [17]. The study examined the chemical and sensory qualities of meat from both green and dry-cured hams, focusing on muscles such as *semitendinosus*, *semimembranosus*, and *biceps femoris*. However, the results did not offer any information on the fatty acid composition of the meat, which is crucial for producing top-quality pork products. Considering the high value of the whole Italian heavy pig production chain and the potential added value of fresh meat showing a quality, if properly matured, that is comparable to the pork meat production (pig slaughtered at 110 kg at approximately 6 months of age), the present study aimed to investigate the effect of immunocastration compared to surgical castration on the chemical composition and fatty acid profile of loins *(longissimus dorsi* muscle) and adipose tissue from Italian male heavy pigs raised for dry-cured ham production.

## 2. Materials and Methods

### 2.1. Ethics Statement

All the procedures included in this study were approved by the Animal Welfare Committee of University of Milan (OPBA_26_2020), in accordance with Directive 2010/63/EU [25].

### 2.2. Subjects and Treatment Groups

The subjects of the present study are part of the work of Pesenti Rossi et al. (2022) [22], who evaluated immunocastration as an alternative to surgical castration in heavy pig production systems. At slaughter, 12 male commercial-hybrid pigs were randomly selected from each treatment group: immunocastration group (IC, *n* = 12) and surgical castration group (SC, *n* = 12). Meat from these carcasses was used for the evaluation of the chemical composition and fatty acid profile of loins (*longissimus dorsi* muscle) and adipose tissue.

The SC pigs (*n* = 12) were surgically castrated at 4 d of age, according to Council Directive 2008/120/EC [26] and standard farming procedures. The IC pigs (*n* = 12) were vaccinated with Improvac^®^ (Zoetis Italia Srl, Roma, Italy), according to the local supplier instructions with a 5-dose regimen. Improvac^®^ was injected subcutaneously behind the base of the ear at 15, 22, 24, 32, and 36 weeks of age (the detailed vaccination protocol is available in Pesenti Rossi et al., 2022 [22]). The pigs in both treatment groups were cared for and managed according to Council Directive 2008/120/EC [26]. The animals received commercial diets based on the nutritional requirements of the growing–fattening phase [27] and on the feeding requirements for the production of Parma Ham (Reg. No 2010/148/EC) [28]. The pigs were slaughtered at the end of the production cycle at 40–41 weeks of age.

### 2.3. Carcass Measurements

The routine abattoir process included carcass weighing and classification, according to Decision 2014/38/EC [29], using the Fat-O-Meter system, which measures backfat (F) and *longissimus dorsi* muscle (M) thickness between the third- and fourth-last rib, 8 cm off the midline of the split carcasses. Through the combination of the two measurements, lean meat content was calculated and expressed as a percentage (%) [30]; the carcasses were classified based on the lean meat content using the European SEUROP system, according to Reg. 2013/1308/EC [31] and Reg. 2017/1182/EC [32]. From each animal, a sample of *longissimus dorsi* muscle and subcutaneous fat was collected between the 10th and 11th rib for meat quality and chemical analysis. The samples were immediately stored at −20 °C.

### 2.4. Chemical Analysis of the Meat and Backfat

#### 2.4.1. Proximate Composition of Meat

Individual samples of loin muscle and backfat were used for moisture, fat, protein, and ash analysis (AOAC Official Methods no. 950.46, 991.36, 981.10 and 920.153, respectively; AOAC, 2003) [33].

#### 2.4.2. Lipid Extraction and FAMEs Analysis

Frozen samples (loin muscle and backfat) from each animal were thawed at room temperature and homogenised. Particularly, lipids were extracted from 10 g of each meat sample using a mix of 2:1 chloroform:methanol and pure water according to the Folch’s procedure [34]. The organic layer was separated and evaporated under vacuum to dryness. The fatty acids (FA) in the lipid extract were trans esterified at room temperature for 10 min with 0.5 M KOH/MeOH to obtain the corresponding methyl esters (FAME). This chemical transformation was carried out [35], checking for the absence of oxidative and degradation reactions that could have an effect on the final fatty acid composition. The FAME were then extracted using n-hexane, and the organic phase was evaporated under vacuum to dryness. Finally, the obtained FAME extract was analysed by gas chromatography (GC) analysis, as described below. In addition, lipids from 5 g of adipose tissue were extracted, and the fatty acids were methylated and analysed as described above for the meat samples. The FAMEs mixture was then resuspended in 20 μL of n-hexane, and 1 μL of the mixture was injected into the chromatographer-Agilent 7890B GC [system with flame ionisation detector/FID and DB-23 (50%-Cyanopropyl)-methylpolysiloxane capillary column (60 m, 0.25 mm i.d., 0.25 μm film thickness)]. The starting temperature was 165 °C and maintained for 3 min; then, the temperature was increased 1 °C per min, arriving at 195 °C where it was held for another 40 min and ending with an additional increase of 10 °C per min, reaching 240 °C, and maintained for 10 min [27]. The carrier gas was hydrogen maintained at a stable pressure of 16.482 psi. Commercially available standard references were used for the identification of the FAME by comparing their retention times. A cluster of 25 FA was detected in this study [36], which corresponded to >98% of the chromatographic peak areas obtained by the GC. The fatty acid data were reported as mg/100 mg of total FA; FA that accounted for <0.1% were not reported. In addition, two health lipid indexes introduced by Ulbricht and Southgate [37] (atherogenic index (IA), thrombogenic index (IT)) were calculated.

### 2.5. Statistical Analysis

Statistical analysis was performed using SPSS 28 (SPSS Inc., Chicago, IL, USA). Statistical significance was accepted at *p* < 0.05. The data were tested for normality and homogeneity of variance using the Kolmogorov–Smirnov and Levene tests, respectively. Since normality and the homoscedasticity of data distribution could not be assumed (*p* < 0.05), differences between the treatment groups (IC vs. SC) for the variables considered in this study (chemical parameters, FA data) were investigated by the non-parametric Mann–Whitney U test.

## 3. Results and Discussion

No significant differences between the IC and SC pigs were observed for any of the parameters monitored at the slaughterhouse reported in Table 1.

Previous studies found similar results for carcass characteristics measured in lighter male pigs (live weight at slaughter ranging from 95 to 125 kg) with slight differences detected between surgically castrated and immunocastrated groups. Particularly, Daza et al. (2016) [23] noted that immunocastration had a minor impact on ham yield in barrow carcasses at slaughter with hams from surgically castrated males exhibiting a slightly higher yield, albeit only tended to statistical significance. Furthermore, no discernible effects were observed on other parameters, such as carcass weight, lean meat content, or backfat thickness. However, contrasting findings were reported by other researchers [6,8,24,38] who observed disparities in fat thickness and lean meat content among barrows (weighing between 107 and 137 kg and aged 23–30 weeks). Surgically castrated males were found to have a higher fat thickness and lower lean meat content compared to immunocastrated males. The authors posited that these differences may be attributed to (a) lower accretion intensity of backfat at the last rib before immunocastration, likely influenced by higher testosterone levels associated with reduced feed intake, and (b) the developmental retardation of the loin muscle, which is involved in posture and appears to be more affected (reduced) during the finishing period in vaccinated pigs compared to ham and shoulder muscles.

In our study, we did not observe such variations, which could potentially be attributed to the greater live weight range (170–180 kg) and extended age range (40–41 weeks) of the animals included. These characteristics closely mirror those of heavy pigs typically raised in Italy for dry-cured ham production.

In addition, we did not observe any influence of the treatment (IC vs. SC) on the chemical composition of the meat (Table 2).

Literature data show different results for the effect of immunocastration vs. surgical castration on the meat characteristic of pigs. Some authors [7,23,24] did not find significant differences in the proximate composition in both *longissimus* (loin) and *semimembranosus* (leg) muscles of surgically castrated and immunocastrated barrows, including the intramuscular fat (IMF) content. On the contrary, Seiquer et al. (2019) [39] and Perez-Ciria et al. (2021) [21] detected a significant difference in the IMF of *longissimus lumborum* and *gluteus medius* muscles between surgically castrated (higher fat) and immunocastrated (lower fat) pigs. These authors proposed that this phenomenon may stem from the behaviour of immunocastrated pigs, resembling that of entire males until the administration of the second vaccine dose that results in elevated testosterone levels that lead to diminished feed intake and, subsequently, reduced fat deposition. Furthermore, they suggested that disparities in genetic background and variations in the vaccination protocol could also exert an influence on meat characteristics.

The level of IMF is fundamental to assure good sensorial quality and high consumer acceptability in pork fresh meat. Generally, we found higher values for the fat content in the loins from heavy pigs (13–14%) than the values found from the other authors in the above-mentioned studies (2–7% of fat) with no differences between the two groups (IC and SC). This could be due to the genetic background of the pigs in our study with a higher body weight at slaughter and a longer production cycle.

Individual and breed genetic variability should be considered. Martinez-Macipe et al. (2016) [40] investigated the effect of immunocastration in pigs from the Valdesequera line (Iberian pigs) reared in a typical extensive system (*montanera*) and slaughtered at an average weight of 156 kg, which is more similar to our case study. These authors found that the IMF content in *longissimus thoracis* of Iberian heavy pigs reached 7–9% fat on average with the highest value recorded for surgically castrated pigs. The authors suggested that meat obtained from immunocastrated males could have a different quality (in terms of meat and sensory characteristics) than meat obtained from surgically castrated pigs. Actually, they found an even higher aroma and flavour of rancidity in meat from immunocastrated pigs, which could be related to a higher susceptibility to oxidation of fatty acids. In predicting pork quality, attention to lipid content and FA profile is crucial, both technologically and nutritionally. These factors influence not only the technological transformation of pork (i.e., a high content of polyunsaturated fatty acids (PUFA) increases the risk of oxidation) but also its nutritional and organoleptic qualities, including intramuscular lipid content, saturated FA content, and the ratio of n-3 to n-6 fatty acids. It is important to note that lipid and FA deposition in pigs is influenced by various factors, such as genotype, sex, age, live weight, environmental temperature, and nutrition [41,42]. These variables play a significant role in shaping the composition of pork tissues. For example, variations in fatty acid profiles can emerge among distinct anatomical regions of the pig, each designated for specific culinary applications. In our study, we examined the loin, rather than the hind leg typically used in the crafting of Italian dry-cured ham, and this should be taken into account in assessments of the nutritional characteristics of a product intended for fresh consumption.

Differences were found in the FA profile of the intramuscular (Table 3) and subcutaneous fat (Table 4).

In our study, the proportion of saturated fatty acids (SFA) in the intramuscular fat was higher in the IC than SC animals (*p* = 0.002), mostly due to the significantly higher proportions of C16:0 (*p* = 0.012) and C18:0 (*p* = 0.008). No corresponding evidence regarding this phenomenon has been encountered in the literature. Nonetheless, from a nutritional perspective, these results are suboptimal due to the detrimental role that saturated fatty acids play in consumer health (i.e., oxidative and inflammatory statuses, predisposition to develop atherosclerotic pathologies and cardiovascular diseases). It is worth mentioning that, while this holds particularly true for medium-chain SFA (12:0–16:0), stearic acid poses fewer concerns as human metabolism is capable of converting it into oleic acid (18:1n-9) through the process of unsaturation [43].

Regarding monounsaturated fatty acids (MUFA), a significantly higher value (*p* < 0.001) was found in loins from the SC animals, mainly due to a significantly higher proportion of oleic acid (C18:1n-9, *p* < 0.001). There is no existing literature indicating specific references to this phenomenon due to immunocastration in fresh pork meat. However, being that oleic acid is one of the primary FA synthesised ex novo during lipogenesis in pigs [23], it could be suggested that immunocastration reduced the lipogenetic activity and consequent MUFA synthesis, delivery, and concentration in the *L. dorsi* muscle of the heavy pigs enrolled in this study.

No significant difference (*p* = 0.12) was observed in the percentage of total PUFA in the loins of the two groups, including n-6 and n-3 FA and their ratio (n-6 to n-3). These results confirm what has been previously reported in the literature [17,19] and provide an interesting insight from a nutritional standpoint. Specifically, IC meat is neither superior nor inferior to SC meat in terms of essential and long-chain FA, which have diverse positive (for n-3) and negative (for n-6) effects on consumer health [44,45]. In pork meat, the main representatives of the n-6 and n-3 series PUFA are linoleic acid (18:2n-6) and α-linolenic acid (18:3n-3), respectively. The method of castration cannot influence the presence of these FA in meat as they are essential fatty acids that, by nature, must be introduced with the diet since mammalian metabolism cannot synthesise them [46].

With regard to the indexes, no differences were detected in the n-6/n-3 ratio between IC and SC in fresh meat (*p* = 0.371), while higher values were found in IC for AI (*p* = 0.003) and TI (*p* = 0.006) at the intramuscular fat level. The ideal n-6 to n-3 ratio for a nutritionally optimal product should be as close to 1:1 as possible [47], whereas in this case, we observed a value of 13:1, which accurately reflects the pigs’ feeding regimen adopted on the farm (i.e., grains and plant sources enriched in linoleic acid). In this regard, linoleic acid was found in IC and SC with a similar concentration, approximately 7–8% of total FAs.

Other than the n-6 to n-3 ratio, thrombogenic and atherogenic indexes are often used to assess the nutritional quality of food products, referring to the relationship between the pro-thrombogenic and/or pro-atherogenic FA (mainly, medium-chain FA) and the anti-thrombogenic and/or anti-atherogenic FA (unsaturated fatty acids) [48]. In the fresh meat of the loins from the heavy pigs enrolled in this study, we observed significant differences between the groups with the SC animals showing more favourable (lower) values for both TI (1.28 in SC and 1.48 in IC) and AI (0.50 in SC and 0.54 in IC). Being that these two indices are directly proportional to the amount of lauric, myristic, and palmitic acid, this result mainly reflected the differences already discussed for SFA amount. Overall, the observed values are comparable to those reported by [49] in loins (0.48–0.52 AI and 1.09–1.21 for TI) from immunocastrated medium–heavy pigs (slaughtered at 142.32 ± 6.8 kg BW, 197 days) of the same genetic types as for PDO production. The slight differences observed between our values and those reported by Minelli et al. (2019) [49] could be imputed to the different age and weight at slaughter, being that these factors are among those affecting the deposition of lipids and their FA composition in pig meat [49].

Few significant differences were evidenced in the adipose tissue. The literature suggests that variations in the lipid profile of adipose tissue become apparent when there is a distinct difference in dorsal fat thickness (>4 mm) [50], which is not the case in our study. Therefore, this finding was unexpected. Although a higher proportion of C18:0 occurred in the IC animals (*p* = 0.017), this was not sufficient to influence the proportion of total SFA. The few differences observed primarily concerned the proportion of MUFA, which was significantly lower in the IC than SC animals (*p* = 0.001) even in this case mostly due to lower C18:1n-9 proportions (subcutaneous: *p* = 0.001). Similar to what was observed in the muscle tissue, oleic acid was found to be lower in the IC pigs even in the adipose tissue. This aligns with the findings of Pérez-Ciria et al. (2021) [21] who analysed the FA composition of backfat in Duroc × (Landrace × Large White) male pigs slaughtered at 106–136 kg of BW, comparing surgical and immunocastrated animals [21]. Similar to what was previously discussed for fresh loins, this result could suggest a reduced or slower lipogenesis induced from immunocastration in heavy pigs enrolled in this study. Literature reports that immunocastrated pigs, until the effective dose (2nd or more), are more similar to entire males, who tend to be leaner and exhibit lower accumulation of de novo synthesized MUFA in lipogenesis processes, especially oleic acid and palmitoleic acid. However, palmitoleic acid (16:1n-7) was found at a lower concentration in backfat from IC pigs. According to the findings of Pauly et al. (2009) [6], in the adipose tissue of IC pigs, the lower quantity of oleic acid was accompanied by a higher amount of stearic acid (18:0), the saturated counterpart FA with 18 carbon atoms [6].

In the adipose tissue, the total PUFA proportion was significantly higher in the subcutaneous fat of the IC animals (*p* = 0.024), mostly due to a higher amount of linoleic acid (C18:2n-6, *p* = 0.023) in the IC pigs compared to SC pigs [6,21,24]. As mentioned earlier, for the results found in loins, linoleic acid is an essential fatty acid abundant in grain and oilseeds, and its amount in pork products derives solely from the diet. A greater accumulation of linoleic acid in the adipose tissue of the IC pigs could potentially indicate an increase in the feed intake consequent to the last vaccination boost, a phenomenon that is well documented in the literature for immunocastrated pigs [6,7,19]. However, further research is needed to understand to which extent the accumulation of linoleic acid is due to the increased feed intake following the last vaccination boost.

Regarding the indexes, no differences were detected in the n-6/n-3 ratio (*p* = 0.194), AI (*p* = 0.818), and TI (*p* = 0.194) calculated in the adipose tissue. Similar to what was observed for fresh meat, these values were comparable to those reported by [49] in the inner and outer layer of backfat (0.48–0.58 AI and 1.09–1.45 for TI) from IC medium–heavy pigs. However, the values reported in our study were slightly higher than those reported by [49], probably due to the effect of a different age and weight at slaughter, which affected FA turnover even in the adipose tissue, as suggested above for the results found in fresh meat.

## 4. Conclusions

The results of our study point out that immunocastration represents an effective alternative to surgical castration for heavy pigs in terms of animal growth and carcass composition; indeed, no significant differences were found for any of the parameters monitored at the slaughterhouse nor for the chemical composition of the meat. On the other hand, some differences were found in the lipid composition of the intramuscular and subcutaneous fat. In particular, it should be noticed that the proportion of saturated fatty acids in the IMF was higher in the immunocastrated pigs, resulting in a suboptimal condition for consumer health. Interestingly, the results obtained by comparing the proportion of MUFA in the two groups of pigs (surgically castrated and immunocastrated) suggested that, as observed in the literature for medium–heavy pigs, the practice of immunocastration could be responsible for a reduced (or slowed-down) lipogenesis even in heavy pigs, a phenomenon highlighted by the significant lower percentages of de novo synthesised MUFA in both muscle and subcutaneous fat of immunocastrated pigs.

## Figures and Tables

**Table 1:** Differences in the parameters monitored at the slaughterhouse between surgically castrated (SC, *n* = 12) and immunocastrated pigs (IC, *n* = 12) expressed as mean ± standard deviation and the number of pigs included in SEUROP classes HU (Heavy, Very good) and HR (Heavy, Good).

Carcass Characteristic	SC	IC	*p*-Value
Live weight, kg	169.97 ± 17.07	175.62 ± 9.53	0.328
Hot carcass weight, kg	145.93 ± 14.66	150.78 ± 8.18	0.328
Cold carcass weight, kg	143.02 ± 14.36	147.77 ± 8.02	0.328
Backfat thickness between 10th and 11th rib, mm	33.11 ± 5.93	30.34 ± 5.54	0.250
*Longissimus dorsi* muscle, mm	58.06 ± 8.23	53.20 ± 8.51	0.169
Lean meat content, %	50.49 ± 2.77	51.64 ± 2.58	0.304
SEUROP, N	HU N = 7	HU N = 9	
	HR N = 6	HR N = 3	

**Table 2:** Chemical composition of meat from surgically castrated (SC, *n* = 12) and immunocastrated pigs (IC, *n* = 12) expressed as mean ± standard deviation.

	SC	IC	*p*-Value
Moisture, %	69.44 ± 3.64	71.14 ± 3.26	0.240
Ash, %	0.94 ± 0.11	0.89 ± 0.14	0.381
Protein, %	15.44 ± 2.07	14.89 ± 1.63	0.474
Fat, %	14.18 ± 1.97	13.09 ± 2.08	0.200

**Table 3:** Fatty acid profile of intramuscular fat (*longissimus dorsi*) from surgically castrated (SC, *n* = 12) and immunocastrated (IC, *n* = 12) pigs expressed as mean ± standard deviation.

Fatty Acids (% of Total FA)	SC	IC	*p*-Value
14:0	1.33 ± 0.10	1.39 ± 0.10	0.180
16:0	24.32 ± 0.79	25.26 ± 0.90	0.012
17:0	0.16 ± 0.03	0.18 ± 0.04	0.152
18:0	14.11 ± 1.58	15.82 ± 1.26	0.008
20:0	0.20 ± 0.02	0.19 ± 0.03	0.179
Total SFA	40.25 ± 2.02	42.98 ± 1.82	0.002
16:1n-7	2.72 ± 0.53	2.60 ± 0.45	0.563
18:1n-9	44.45 ± 1.14	40.84 ± 1.95	0.000
18:1n-7	2.95 ± 0.42	2.85 ± 0.49	0.583
20:1n-9	0.86 ± 0.09	0.80 ± 0.07	0.066
Total MUFA	51.00 ± 1.55	47.09 ± 2.63	0.000
18:2n-6	7.53 ± 0.84	8.51 ± 1.94	0.124
18:3n-3	0.25 ± 0.03	0.27 ± 0.09	0.488
20:2n-6	0.37 ± 0.05	0.41 ± 0.11	0.275
20:3n-6	0.09 ± 0.02	0.12 ± 0.03	0.028
20:3n-3	0.34 ± 0.10	0.43 ± 0.13	0.067
Total PUFA	8.75 ± 0.97	9.93 ± 2.21	0.105
Total n-3	0.60 ± 0.11	0.71 ± 0.14	0.051
Total n-6	8.14 ± 0.91	9.22 ± 2.10	0.119
n-6/n-3	13.73 ± 2.02	13.01 ± 1.84	0.371
AI	0.50 ± 0.03	0.54 ± 0.04	0.003
TI	1.28 ± 0.11	1.42 ± 0.11	0.006

SFA = saturated fatty acids; MUFA = monounsaturated fatty acids; PUFA = polyunsaturated fatty acids; AI = atherogenic index; TI = thrombogenic index.

**Table 4:** Fatty acid profile of subcutaneous fat (10th–11th rib) from surgically castrated (SC, *n* = 12) and immunocastrated (IC, *n* = 12) pigs expressed as mean ± standard deviation.

Fatty Acids (% of Total FA)	SC	IC	*p*-Value
14:0	1.48 ± 0.14	1.39 ± 0.09	0.066
16:0	27.78 ± 1.18	27.57 ± 0.94	0.628
17:0	0.29 ± 0.05	0.32 ± 0.06	0.161
18:0	20.08 ± 1.93	21.64 ± 0.80	0.017
20:0	0.22 ± 0.02	0.22 ± 0.03	0.763
Total SFA	50.00 ± 2.33	51.30 ± 1.01	0.091
16:1n-7	1.30 ± 0.14	1.16 ± 0.10	0.010
18:1n-9	35.69 ± 2.09	32.86 ± 1.52	0.001
18:1n-7	1.06 ± 0.14	0.99 ± 0.06	0.120
20:1n-9	0.69 ± 0.17	0.63 ± 0.14	0.342
Total MUFA	38.74 ± 2.39	35.64 ± 1.62	0.001
18:2n-6	9.97 ± 1.43	11.66 ± 1.92	0.023
18:3n-3	0.49 ± 0.09	0.57 ± 0.11	0.084
20:2n-6	0.45 ± 0.06	0.48 ± 0.06	0.249
20:3n-3	0.15 ± 0.03	0.15 ± 0.02	0.755
Total PUFA	11.26 ± 1.59	13.07 ± 2.09	0.027
Total n-3	0.65 ± 0.12	0.73 ± 0.13	0.132
Total n-6	10.61 ± 1.49	12.34 ± 1.97	0.024
n-6/n-3	16.44 ± 1.18	16.99 ± 0.83	0.194
AI	0.68 ± 0.06	0.68 ± 0.04	0.818
TI	1.88 ± 0.18	1.96 ± 0.09	0.194

SFA = saturated fatty acids; MUFA = monounsaturated fatty acids; PUFA = polyunsaturated fatty acids; AI = atherogenic index; TI = thrombogenic index.

## Data Availability

The data presented in this study are available upon request to the corresponding author.
